# Improved β-cell function leads to improved glucose tolerance in a transgenic mouse expressing lipoprotein lipase in adipocytes

**DOI:** 10.1038/s41598-022-26995-1

**Published:** 2022-12-24

**Authors:** Hasiyet Memetimin, Beibei Zhu, Sangderk Lee, Wendy S. Katz, Philip A. Kern, Brian S. Finlin

**Affiliations:** 1 Division of Endocrinology, and the Barnstable Brown Diabetes and Obesity Center, Department of Medicine, University of Kentucky, Lexington, KY USA; 2 Department of Pharmacology and Nutritional Sciences, University of Kentucky, Lexington, KY USA

**Keywords:** Endocrine system and metabolic diseases, Transcription

## Abstract

Lipoprotein lipase (LPL) hydrolyzes the triglyceride core of lipoproteins and also functions as a bridge, allowing for lipoprotein and cholesterol uptake. Transgenic mice expressing LPL in adipose tissue under the control of the adiponectin promoter (*AdipoQ-LPL*) have improved glucose metabolism when challenged with a high fat diet. Here, we studied the transcriptional response of the adipose tissue of these mice to acute high fat diet exposure. Gene set enrichment analysis (GSEA) provided mechanistic insight into the improved metabolic phenotype of AdipoQ-LPL mice. First, the cholesterol homeostasis pathway, which is controlled by the SREBP2 transcription factor, is repressed in gonadal adipose tissue AdipoQ-LPL mice. Furthermore, we identified SND1 as a link between SREBP2 and CCL19, an inflammatory chemokine that is reduced in AdipoQ-LPL mice. Second, GSEA identified a signature for pancreatic β-cells in adipose tissue of AdipoQ-LPL mice, an unexpected finding. We explored whether β-cell function is improved in AdipoQ-LPL mice and found that the first phase of insulin secretion is increased in mice challenged with high fat diet. In summary, we identify two different mechanisms for the improved metabolic phenotype of AdipoQ-LPL mice. One involves improved adipose tissue function and the other involves adipose tissue—pancreatic β-cell crosstalk.

## Introduction

Lipoprotein lipase (LPL) is highly expressed in adipose tissue and promotes the uptake of lipid for storage^1^. We have developed a mouse model using the adiponectin promoter to increase LPL expression in adipose tissue^2^. These mice (AdipoQ-LPL) have improved glucose metabolism when challenged with a high fat diet^2^. Our initial characterization of the mouse revealed that ectopic lipids were not reduced in either liver or skeletal muscle. This was likely due to the fact that the adiponectin promoter that we utilized only increased *LPL* mRNA 1.2 fold over endogenous *LPL* levels^2^. However, gene expression was altered in the perigonadal fat of male mice, in a manner suggesting that AdipoQ-LPL mice have less adipose dysfunction. For instance, the mRNA levels of adiponectin and other peroxisome proliferator-activated receptor (PPARγ)-regulated genes were higher in AdipoQ-LPL mice with trends for reduced inflammatory gene expression at the end of a high fat diet induced obesity study^2^.

Changes in adipose tissue gene expression are observed in as little as 24-h after high fat diet feeding of mice^3^, and these changes are thought to initiate the development of adipose dysfunction^4,5^. One mechanism postulates that the increased hypertrophy of adipose tissue causes hypoxia and the induction of collagens which cause adipocyte death. This in turn leads to macrophage accumulation, inflammation, and adipose dysfunction^5^. Additional pathways activated by high fat feeding include endoplasmic reticulum stress; transforming growth factor-β (TGFβ) and fibrosis; and sterol regulatory element-binding protein 2 (SREBP2)^3,6,7^. Thus, identifying changes in gene expression that occur early during an acute high fat diet challenge is important for understanding mechanisms that cause the development of adipose dysfunction.

In this study, we characterize the body composition and energy expenditure of AdipoQ-LPL mice during the chow to high fat diet transition. We then identify changes in gene expression and the pathways affected in the gonadal fat of AdipoQ-LPL mice that occur early in the course of high fat diet feeding using gene set enrichment analysis (GSEA) of hallmark gene sets and other approaches. These analyses of gene expression led us to evaluate several interesting mechanisms for the improved metabolic phenotype of the AdipoQ-LPL mouse model.

## Results

### Study design, body composition of the mice, and indirect calorimetry

The AdipoQ-LPL mouse model has improved glucose metabolism when challenged with a high fat diet^2^. Additionally, the mice have increased food intake and increased energy expenditure after 12-weeks of high fat diet^2^. To better understand these changes that occur after a chronic high fat diet, we characterized the initial response of the mice to high fat diet by indirect calorimetry. After a 1-week acclimation period, mice were placed in calorimetry chambers and studied for 1-week on chow and 1-week on high fat diet. Three days after the mice were removed from the calorimetry chambers (10-days total on HFD), the mice were euthanized and tissues collected for gene expression studies. An additional cohort was maintained on chow, but not characterized by microarray.

Table 1 shows the body composition of all of the mice in the study before being placed into acclimation chambers. The AdipoQ-LPL mice had a slightly higher (0.5 g) fat mass than the control mice (Table 1; P = 0.04); otherwise, the two groups of mice were similar. The mice were then split into two groups; one group was switched to high fat diet after 1-week in calorimetry chambers and the other was maintained on chow diet. The acute high fat diet resulted in an increase in fat mass in both groups of mice as expected (Table 1); there were no differences between the control and AdipoQ-LPL mice in weight gain, fat mass gain, or lean mass gain (Table 1). Although not statistically significant, the AdipoQ-LPL mice gained 0.4 g of fat more than the control mice over 10-days of chow feeding (Table 1; P = 0.12). When the calorimetry data were analyzed for the group that was switched from chow to HFD in calorimetry chambers, there were no differences between the control and AdipoQ-LPL mice in food intake, resting respiratory exchange ratio, or resting energy expenditure.

**Table 1:** ECHO MRI characterization of mice.

Parameter	Control	AdipoQ-LPL	*P*
**All mice before the study**^**a**^			
Weight (g)	26.4 ± 0.5	26.4 ± 0.3	0.90
Lean mass (g)	19.9 ± 0.4	19.3 ± 0.2	0.23
Fat mass (g)	2.8 ± 0.2	3.3 ± 0.2	0.04
**Mice challenged with HFD**^**b**^			
Weight (g)	27.8 ± 0.6	26.4 ± 0.5	0.06
Initial lean mass (g)	20.9 ± 0.5	19.6 ± 0.4	0.03
Final lean mass (g)	20.7 ± 0.5	19.5 ± 0.3	0.03
Delta lean mass (g)	−0.2 ± 0.3	−0.1 ± 0.2	0.70
Initial fat mass (g)	3.0 ± 0.3	3.2 ± 0.3	0.62
Final fat mass (g)	7.7 ± 0.5	7.4 ± 0.4	0.61
Delta fat mass	4.7 ± 0.3	4.2 ± 0.3	0.29
**Mice maintained on chow**^**c**^			
Initial weight (g)	25.2 ± 2	26.3 ± 1.6	0.13
Initial lean mass(g)	18.9 ± 1.5	19.1 ± 0.8	0.71
Final lean mass (g)	19.7 ± 1.3	19.6 ± 0.9	0.77
Delta lean mass (g)	0.8 ± 0.7	0.5 ± 0.5	0.20
Initial fat mass (g)	2.6 ± 0.5	3.5 ± 0.9	0.01
Final fat mass (g)	3.3 ± 0.8	4.6 ± 1.7	0.02
Delta fat mass (g)	0.7 ± 0.4	1.1 ± 0.9	0.12

^a^AdipoQ-LPL transgenic male mice and their littermate controls (age 9–9.5 weeks) were characterized by ECHO-MRI before and after an acute high fat diet challenge in calorimetry chambers; a group of mice was maintained on chow as a control group. These data are for the entire cohort (control n = 25; AdipoQ-LPL n = 23) before the study. Data are represented as means ± standard error of the mean.

^b^AdipoQ-LPL transgenic male mice and their littermate controls were characterized in a 1-week chow and 1-week high fat diet (60% cal fat) feeding study in calorimetry chambers (n = 12 control and n = 12 AdipoQ-LPL per group). Data are represented as means ± standard error of the mean.

^c^AdipoQ-LPL transgenic male mice and their littermate controls were maintained on chow (n = 13 control and n = 11 AdipoQ-LPL per group). Data are represented as means ± standard error of the mean.

#### Microarray

We next performed a microarray analysis on the perigonadal fat from 12 control and 12 AdipoQ-LPL male mice from the 10-day acute high fat diet challenge. Genes with a 1.25-fold difference in expression and P < 0.02 are listed in Table S1. Functional annotation analysis of these genes using NIH David^8,9^ indicated that two KEGG pathways were enriched in this gene list. The steroid biosynthesis pathway was the most significantly enriched pathway (P = 6e^−5^). The enzymes NAD(P) dependent steroid dehydrogenase-like (*Nsdhl*); lanosterol synthase (*Lss*); cytochrome P450 family 51 (*Cyp51*), and squalene epoxidase (*Sqle*) were the genes in the steroid biosynthesis KEGG pathway, and all four genes were lower in AdipoQ-LPL mice (Table S1). Further analysis of the gene list (Table S1) showed that two other genes involved in cholesterol homeostasis, the low density lipoprotein receptor (*Ldlr*) and insulin induced gene 1 (*Insig1*), are also repressed in AdipoQ-LPL mice. Together, these data suggest that cholesterol biosynthesis is down-regulated in the adipose of AdipoQ-LPL mice; this could be due to increased lipoprotein core uptake by the LPL, which has been demonstrated to occur in other tissues^10–16^. The PPAR signaling KEGG pathway was also detected (P = 0.05), which was expected since we previously detected a PPAR gene signature in AdipoQ-LPL mice fed a high fat diet for 16-weeks^2^. Phosphoenolpyruvate carboxykinase 1 (*Pck1*) was induced in AdipoQ-LPL mice (Table S1); in addition, the microarray indicated that adiponectin is induced in AdipoQ-LPL mice. The shuttle for transfer of acetyl groups from the mitochondria to the cytosol BIOCARTA pathway was detected (P = 0.05). This pathway is involved in lipid biosynthesis, and the two genes in this pathway, malic enzyme 1 (*Me1*) and ATP citrate lyase (*Acly*), are repressed in AdipoQ-LPL mice (Table S1). In addition, Solute Carrier Family 25 Member 10 (*Slc25a10*), which is important for citrate transport from the mitochondria to the cytosol and lipogenesis^17^ was also repressed in AdipoQ-LPL mice (Table S1). Thus, key genes in lipid and cholesterol biosynthesis are repressed in AdipoQ-LPL mice, consistent with increased expression of LPL by the transgene.

**Figure 1:**
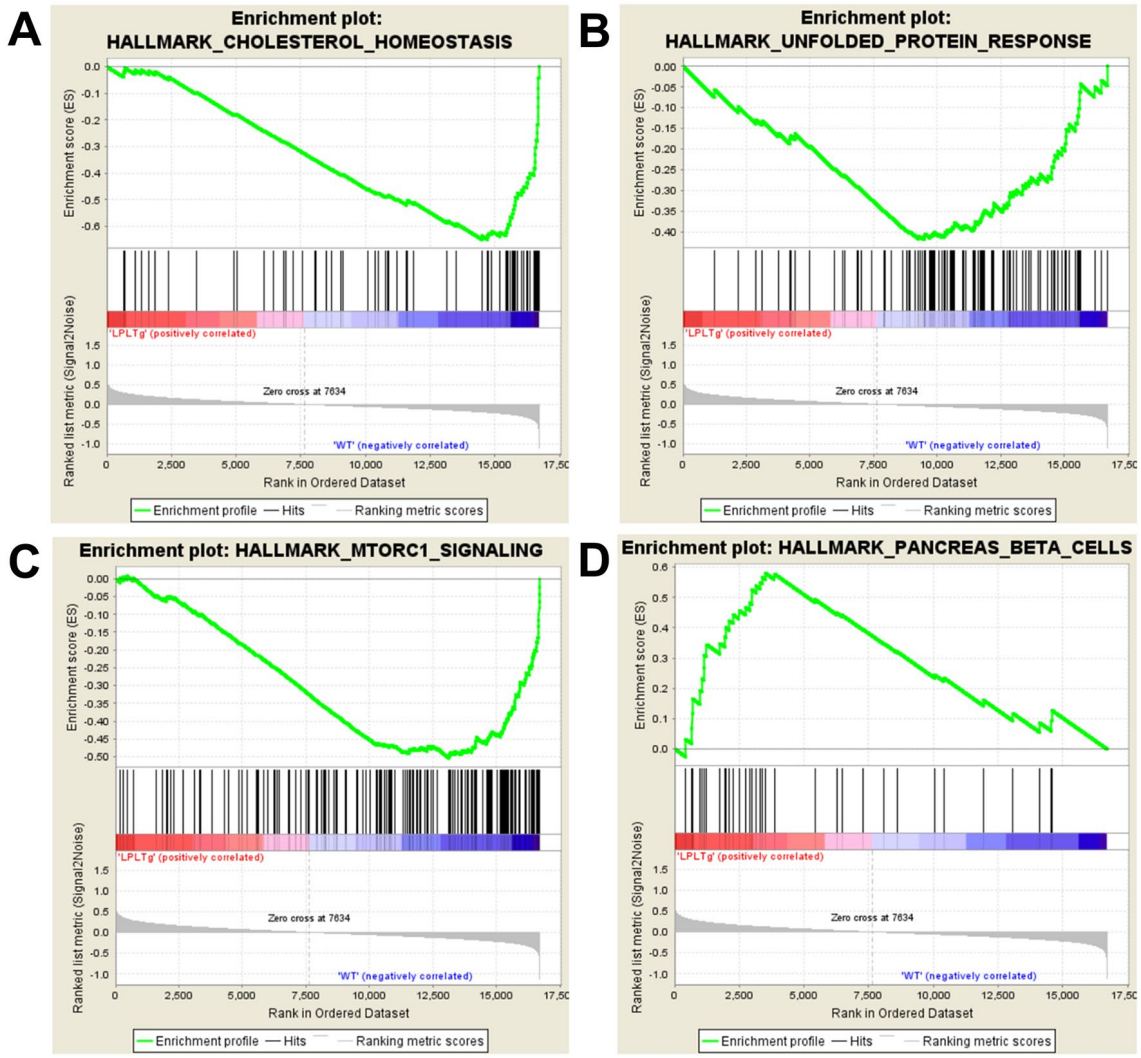
Enrichment plots of hallmark gene sets enriched and repressed in perigonadal adipose tissue of male AdipoQ-LPL mice. We used Affymetrix Mouse 2.0 microarrays to quantify gene expression in perigonadal adipose tissue and analyzed the entire set of data by gene set enrichment analysis to identify hallmark gene sets enriched or repressed in the AdipoQ-LPL mice (n = 12). (**A**–**D**) Enrichment plots for gene sets repressed and enriched in AdipoQ-LPL mice.

To gain further insight into the transcriptional response of the mice, we performed gene set enrichment analysis of hallmark gene sets on all of the microarray data^18–20^. In addition to suppression of the cholesterol homeostasis pathway, this analysis identified several pathways that contribute to adipose dysfunction are repressed in AdipoQ-LPL mice including the unfolded protein response (endoplasmic reticulum stress) and mammalian target of rapamycin complex 1 (MTORC1 signaling) (Table 2); example enrichment plots are shown in Fig. 1. The TGFβ signaling and hypoxia gene sets had trends for reduction (Table 2). These pathways are upregulated early in the course of the development of adipose dysfunction by high fat feeding and are intertwined with each other. Furthermore, these pathways are linked to inflammation and fibrosis by mechanisms that are incompletely understood. An unexpected finding was the enrichment of genes involved in pancreatic β-cell development and function in the AdipoQ-LPL mice (Table 2), suggesting a possible link between adipose tissue and pancreatic β-cell function, contributing to improved glucose homeostasis. GSEA analysis thus suggested two mechanisms for the improved glucose homeostasis of the AdipoQ-LPL mice. One mechanism involves an effect on adipose tissue and the other involves adipose tissue—pancreatic β-cell cross talk.

**Table 2:** Enrichment of hallmark gene sets in adipose tissue.

Gene sets down in AdipoQ-LPL mice					
Name	SIZE	ES	NES	NOM P-val	FDR q-val
HALLMARK_CHOLESTEROL_HOMEOSTASIS	72	− 0.65	− 1.78	0.002	0.127
HALLMARK_UNFOLDED_PROTEIN_RESPONSE	109	− 0.42	− 1.65	0.030	0.270
HALLMARK_MTORC1_SIGNALING	185	− 0.50	− 1.59	0.042	0.281
HALLMARK_TGF_BETA_SIGNALING	54	− 0.40	− 1.46	0.085	0.417
HALLMARK_UV_RESPONSE_UP	143	− 0.29	− 1.27	0.096	0.355
HALLMARK_MYC_TARGETS_V1	175	− 0.44	− 1.58	0.100	0.235
HALLMARK_APICAL_JUNCTION	198	− 0.33	− 1.29	0.101	0.431
HALLMARK_HYPOXIA	191	− 0.32	− 1.28	0.105	0.385
HALLMARK_MYOGENESIS	195	− 0.38	− 1.28	0.106	0.413
**Gene sets up in AdipoQ-LPL mice**					
HALLMARK_PANCREAS_BETA_CELLS	37	0.58	1.69	0.008	0.137
HALLMARK_COAGULATION	131	0.44	1.39	0.061	0.578

The microarray data for adipose tissue gene expression was analyzed by GSEA to evaluate hallmark gene sets downregulated or upregulated in AdipoQ-LPL mice. The name of the pathway, number of genes (SIZE), Enrichment and normalized enrichment scores, Nominal P values and false discovery rate q-values generated by GSEA are indicated.

#### Suppression of the cholesterol homeostasis pathway and identification of SND1 as a link between SREBP2 and inflammation in adipose tissue

**Figure 2:**
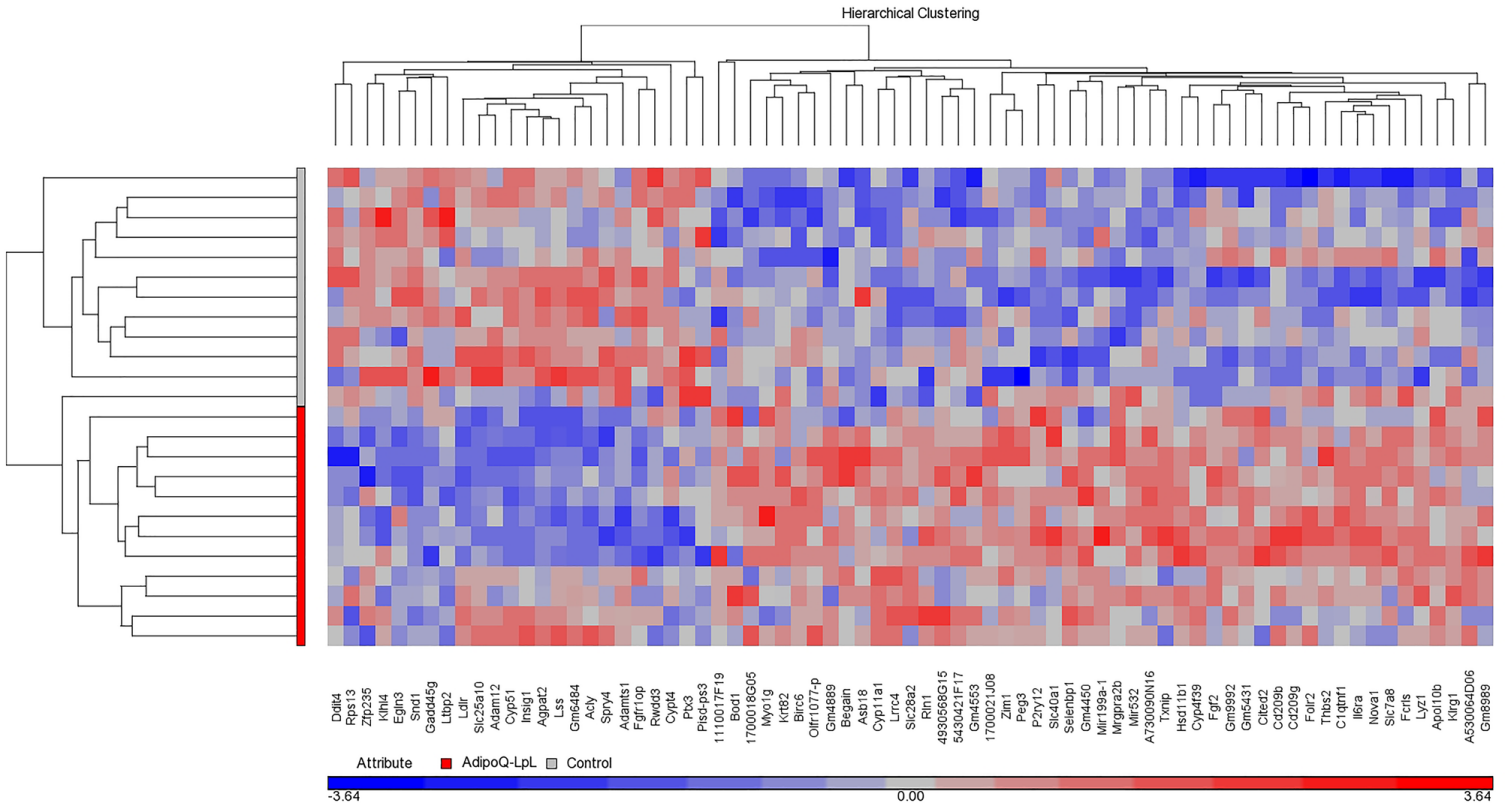
Hierarchical clustering of gene expression. The heat map was generated with Partek using genes with a P value less than 0.01 and a 1.25-fold change in expression.

Hierarchical cluster analysis indicated that SND1 was clustered with genes involved in cholesterol biosynthesis (Fig. 2), suggesting that SND1 is regulated by SREBP2. SND1 binds RNA and regulates transcription, splicing, mRNA stability, and miRNA biosynthesis. SND1 is conserved from plants to mammals, and one of its primary functions is to protect against stress by regulating cellular stress responses. As an oncogene in hepatic carcinoma, SND1 enhances inflammation and fibrosis^21,22^, suggesting that SND1 could be an important link between SREBP2 and cellular stress responses including inflammation. Suppression of SND1 could thus contribute to the improved transcriptional response of the AdipoQ-LPL mouse.

**Figure 3:**
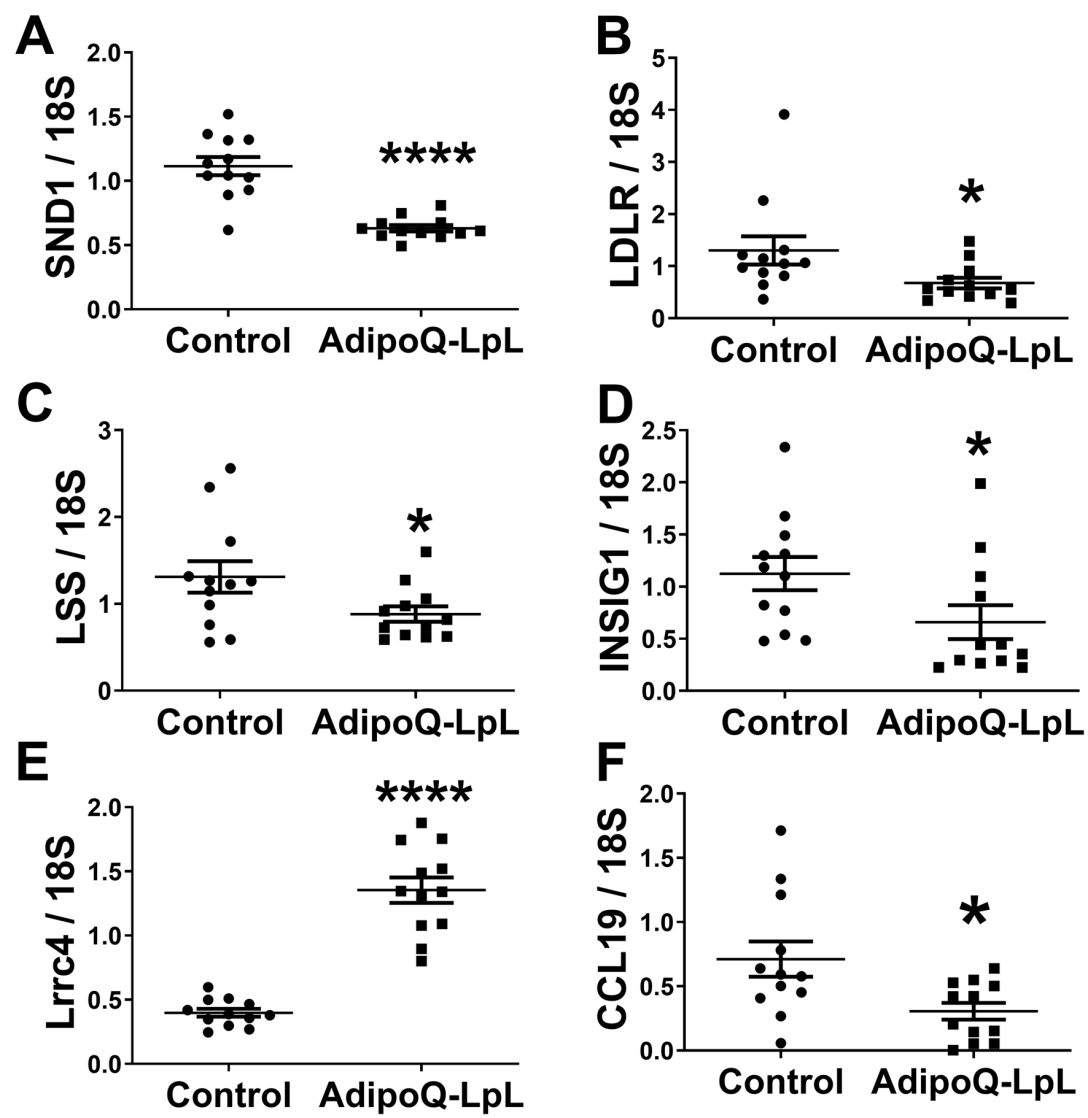
Confirmation of the microarray results. mRNA expression in perigonadal fat of control and AdipoQ-LPL mice on chow or high fat diet (10-days) was measured by real-time RT-PCR. The data are represented as the mean ± SEM of gene expression normalized to 18S (n = 12). mRNA expression of (**A**) SND1, (**B**) LDLR, (**C**) LSS, (**D**) INSIG1, (**E**) Lrrc4, and (**F**) CCl19 is indicated (*P < 0.05; ****P < 0.0001; two-tailed unpaired Student’s t-test). Data for *Ccl19* is log10 transformed.

We verified the microarray results with real time RT PCR. SND1 mRNA was repressed in perigonadal adipose tissue of AdipoQ-LPL mice (Fig. 3A; P < 0.0001). As shown in Fig. 3B–D, *Ldlr*, *Lss* and *Insig1*, which are SREBP2-regulated genes, were all lower in AdipoQ-LPL mice than control mice (P < 0.05). When we examined the *Snd1* gene structure, we found that Leucine Rich Repeat Containing 4 (*Lrrc4*) is nested in an intron of SND1 on the opposite strand^23^. *Lrrc4* mRNA is higher in AdipoQ-LPL mice (Fig. 3E; P < 0.0001). This is the first report of the *Snd1* and *Lrrc4* genes being differentially regulated in vivo, a common phenomenon for nested genes^23^. In fact, these two nested genes had by far the best two P values in this study (Table S1). Finally, although genes become nested by random, it is interesting to note that SND1 is an oncogene and Lrcc4 is a tumor suppressor.

Because of the potential connection between SND1 and inflammation, we evaluated inflammatory gene expression. Inflammation is just beginning to develop at 10-days of high fat feeding^3^; therefore we did not expect to find a large number of inflammatory genes. However, in Table S1, we identified chemokine (C–C motif) ligand 19 (*Ccl19*; macrophage inflammatory protein 3b), a chemokine for dendritic cells, which promote adipose inflammation^24^, and confirmed that it was repressed in AdipoQ-LPL mice (Fig. 3F; P<0.05).

### SND1 mRNA and protein expression and regulation in adipocytes

**Figure 4:**
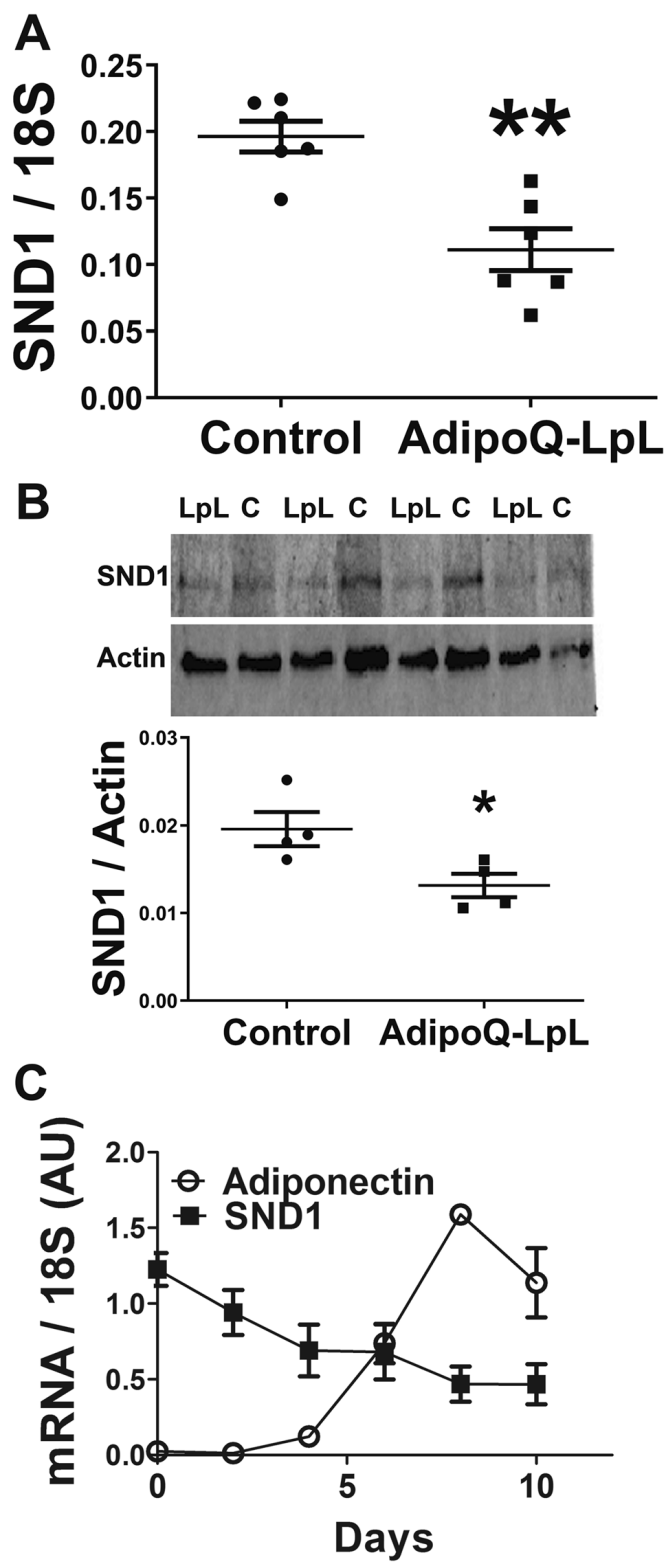
SND1 mRNA and protein expression in adipocytes. (**A**) SND1 mRNA expression was measured in the floating adipocytes obtained by collagenase digestion of adipose tissue from control and AdipoQ-LPL mice high fat diet (10-days). The data are represented as the mean ± SEM (n = 6; **P < 0.01; two-tailed unpaired Student’s t-test). (**B**) SND1 and actin were immunoblotted in lysates of floating adipocytes from control (**C**) and AdipoQ-LPL (LpL) mice on the high fat diet (10-days). The ratio of SND1 to actin was calculated, and the data are represented as the mean ± SEM (n = 4; *P < 0.05; two-tailed unpaired Student’s t-test). (**C**) *Snd1* or *AdipoQ* mRNA expression during a time course of 3T3-L1 adipocyte differentiation (n = 3).

To identify cell types where *Snd1* is expressed in adipose tissue and regulated by LPL, we collagenase digested adipose tissue and measured *Snd1* in the floating adipocytes. *Snd1* mRNA was significantly repressed in the AdipoQ-LPL mouse floating adipocytes (Fig. 4A; P < 0.05). *Snd1* was expressed in the stromal vascular fraction, and there was a trend for lower expression in the AdipoQ-LPL mice (P = 0.07; not shown). Next, we confirmed that SND1 protein levels were reduced in floating adipocytes from the AdipoQ-LPL mouse (Fig. 4B; P < 0.05; images of the uncropped gels are in supplemental Fig. 1). Finally, we characterized *Snd1* mRNA expression during a time course of differentiation of 3T3-L1 cells into adipocytes. *Adiponectin* expression was low in preadipocytes and increased during the time course of differentiation as expected. *Snd1* mRNA was expressed during all stages of differentiation (Fig. 4C) as previously observed^25^. Thus, *Snd1* is expressed in adipocytes, reduced in AdipoQ-LPL mice, and 3T3-L1 adipocytes are a suitable model for in vitro studies of SND1.

**Figure 5:**
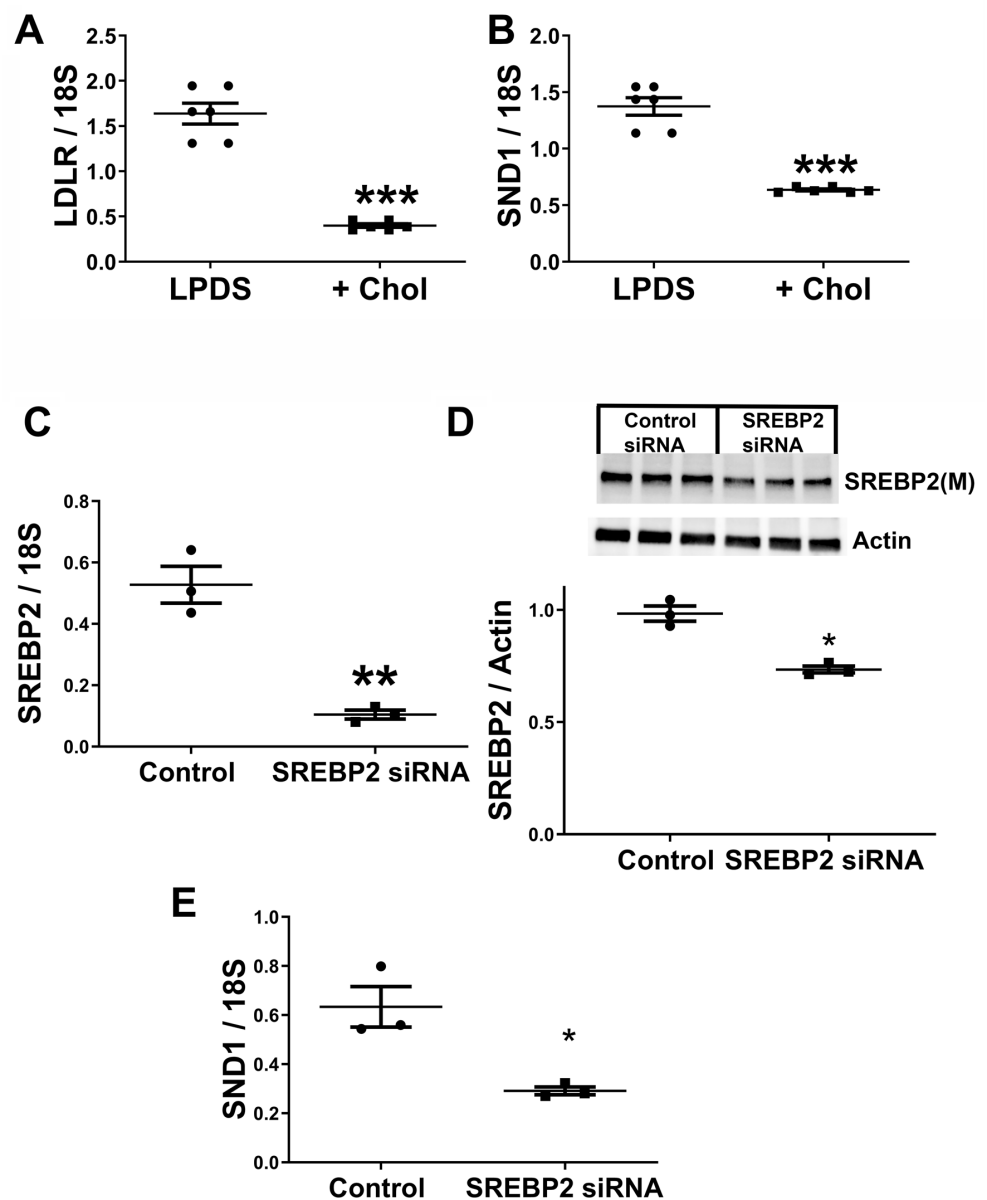
SREBP2 regulates *Snd1* mRNA expression. (**A**,**B**) Differentiated 3T3-L1 adipocytes were treated with lipoprotein deficient serum containing media (LPDS) for 24-h. The cells were then incubated in LPDS or LPDS with cholesterol (Chol) as indicated. Real-time RT-PCR was used to measure the gene expression of *Ldlr* and *Snd1*. Next, differentiated 3T3-L1 adipocytes were treated with control (scrambled) or SREBP2 siRNA. (**C**) SREBP2 mRNA expression was measured. **(D**) SREBP2 (M:mature form) and actin were immunoblotted and quantified. (**E**) SND1 mRNA expression was determined. The data are represented as the mean ± SEM (n = 6 (A and B) or n = 3 (**C**–**E**); *P < 0.05; **P < 0.01; ***P < 0.001 two-tailed unpaired Student’s t-test).

Since *Snd1* clustered with genes in the cholesterol homeostasis pathway, we determined the effects of cholesterol depletion and reduction of SREBP2 by siRNA on *Snd1* mRNA expression in adipocytes. We treated differentiated 3T3-L1 adipocytes with lipoprotein deficient serum (LPDS) containing compactin and a low level of mevalonate to prevent toxicity; cholesterol was added to the same media to inhibit SREBP2. Addition of cholesterol to the media repressed *Ldlr* mRNA expression as expected, and *Snd1* mRNA expression was also repressed (Fig. 5A,B; P < 0.001), suggesting that SREBP2 positively regulates SND1 expression in adipocytes. Next, we used siRNA to reduce SREBP2 in differentiated 3T3L1 cells. Treatment of 3T3L1 cells with SREBP2 siRNA reduced SREBP2 mRNA levels (Fig. 5C; P < 0.01) and SREBP2 protein (mature) levels (Fig. 5D; P < 0.05; images of the uncropped gels are in supplemental Fig. 2). We note that we did not detect the parental form of SREBP2 likely due to the use of serum free medium in this experiment (see full blots in supplemental Fig. 2). Reducing SREBP2 with siRNA reduced SND1 mRNA levels (Fig. 5E; P < 0.05).

### SND1 promotes inflammatory cytokine and chemokine expression in adipocytes

**Figure 6:**
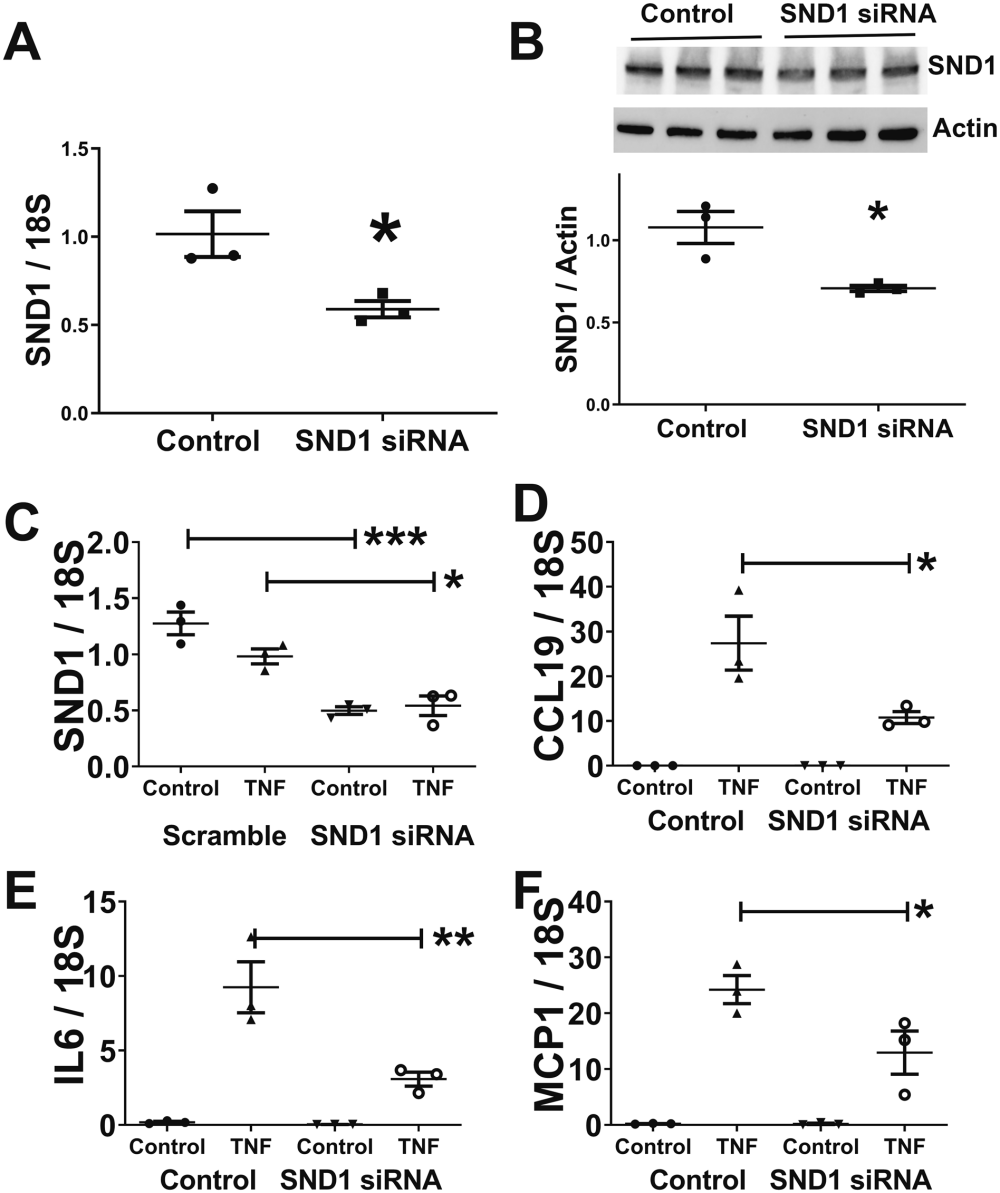
SND1 promotes inflammatory gene expression in adipocytes. (**A**,**B**) Differentiated 3T3-L1 adipocytes were treated with control (scrambled) or *Snd1* siRNA as indicated, and *Snd1*1 mRNA expression and protein expression were determined. (**C**–**F**) Differentiated 3T3-L1 adipocytes were treated with control or Snd1 siRNA as indicated. Next, they were treated with 20 ng/mL TNFα for 6-h. The mRNA expression of (**C**) SND1, (**D**) CCL19, (**E**) IL6, and (**F**) MCP1 was determined with real time RT-PCR. The data are represented as the mean ± SEM (n = 3; *P < 0.05; **P < 0.01; ***P < 0.01; one way ANOVA with Tukey post hoc test); the data for SND1 is log transformed.

SREBP2 promotes inflammation, but the precise mechanism is not known. SND1 promotes nuclear factor kappa-light-chain-enhancer of activated B cells (NFκB) activity in hepatic carcinoma^22^, and could thus be a link between SREBP2 and inflammation in adipocytes. Since activation of the NFκB pathway has been strongly implicated in the development of adipose inflammation^26,27^, we determined whether reducing SND1 expression would inhibit the induction of inflammatory cytokines that are known to be regulated by NFκB in differentiated 3T3-L1 adipocytes. We also evaluated *Ccl19* since it is an inflammatory chemokine^24,28^ that was repressed 1.4-fold in AdipoQ-LPL mice (Table S1; P = 0.013), which was confirmed by real time RT PCR (Fig. 3F; P < 0.05). We treated 3T3-L1 adipocytes with control (scramble) siRNA or SND1 siRNA and 48-h later treated them with tumor necrosis factor-α (TNFα) to induce an inflammatory response. Treatment of differentiated 3T3L1 cells with SND1 siRNA significantly reduced SND1 mRNA and protein levels (Fig. 6A,B; P < 0.05; images of the uncropped gels are in supplemental Fig. 3). Next, we determined whether lowering SND1 with siRNA would reduce inflammatory gene expression in response to TNFα treatment. Interleukin 6 (*IL6*) and monocyte chemoattractant protein 1 (*MCP1*) were evaluated as classical inflammatory genes, and Ccl19 was evaluated because it was shown to be lower in adipose tissue of AdipoQ-LpL mice and is also an inflammatory gene. *Ccl19 IL6*, and *MCP1* were induced by TNFα and repressed in *Snd1* siRNA in 3T3-L1 adipocytes (Fig. 6B–D; P < 0.05).

### SND1 regulates Ccl19 splicing

**Figure 7:**
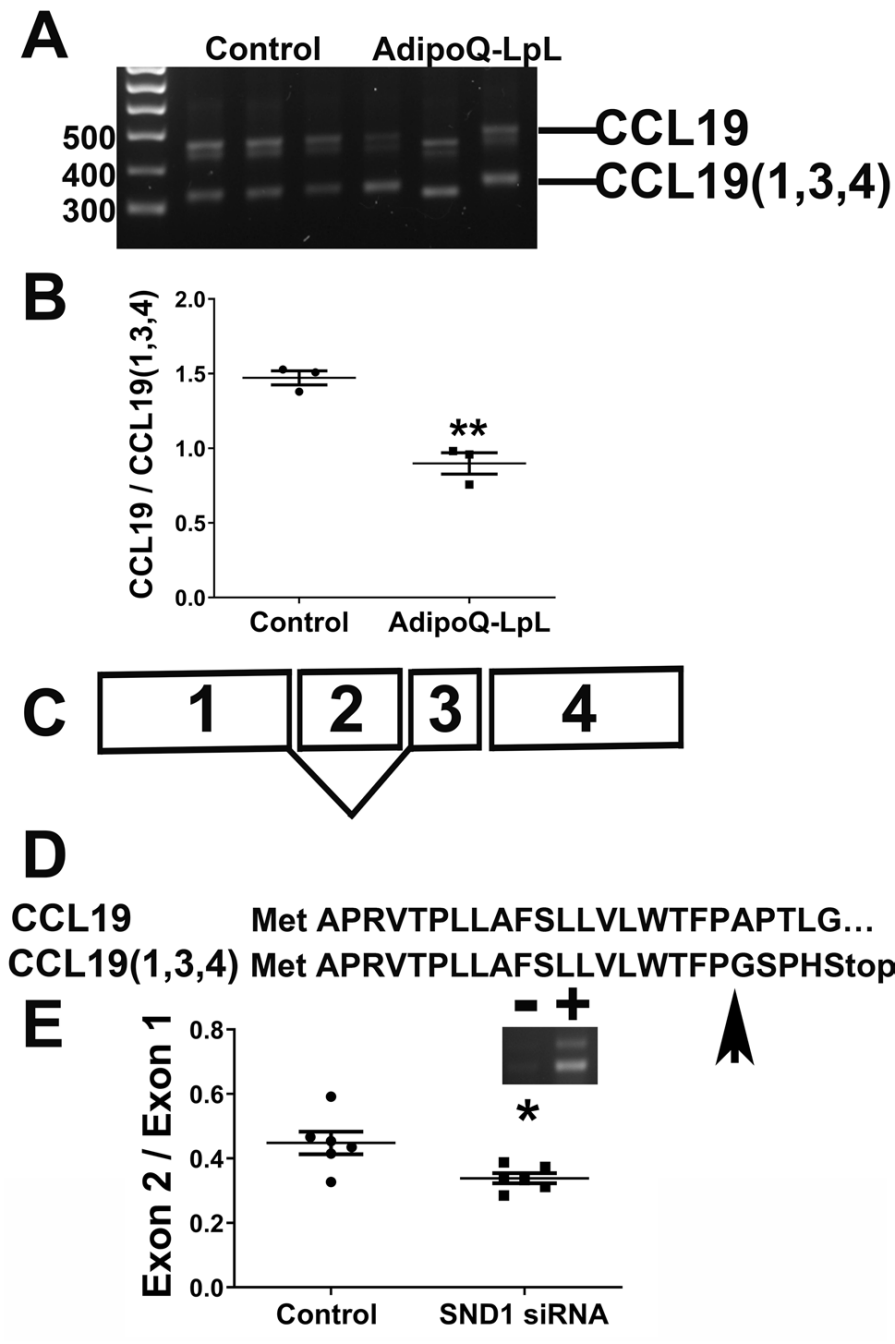
SND1 regulates *Ccl19* splicing. (**A**) RNA from the adipose tissue of control or AdipoQ-LPL mice was subjected to PCR with primers located in exon 1 and 4 of *Ccl19*. The PCR products were resolved on an agarose gel and then sequenced. The positions of full-length *Ccl19* and the shorter isoform, which was found to lack exon 2 by DNA sequencing, are indicated. (**B**) The ratio of full-length *Ccl19* to *Ccl19* (1,3,4) was calculated. The data are represented as the mean ± SEM (n = 3; **P < 0.01; two-tailed unpaired Student’s t-test). (**C**) A diagram showing the four exons of *Ccl19* and alternative usage of exon 2. (**D**) Translation of the full-length and *Ccl19* (1,3,4) reveals that when exon 2 is missing, the reading frame shifts and a premature stop codon is present. The arrow indicates the position of the amino acid affected by the frame shift. (**E**) Inset: induction of two *Ccl19* isoforms in 3T3-L1 adipocytes by TNFα. The ratio of exon 2 to exon 1 in 3T3-L1 adipocytes treated with scrambled or *Snd1* siRNA and the TNFα were calculated by real time RT-PCR with exon specific primers and *Ccl19* plasmid DNA to construct the standard curve. The data are represented as the mean ± SEM (n = 6; *P < 0.05; two-tailed unpaired Student’s t-test).

SND1 regulates mRNA splicing by promoting the inclusion of exons with weak splice sites^29^; therefore, we analyzed the array data for gene splicing. This revealed a significant number of genes with alternative splicing (Table S2) including *Ccl19*. The *Ccl19* gene has 4 exons; the microarray revealed that exons 2 and 3 of *Ccl19* are reduced in AdipoQ-LPL mice, suggesting that shorter isoforms exists. We designed PCR primers in exons 1 and 4 that would generate distinct PCR products if exons 2 or 3 are not in *Snd1* mRNA in adipose tissue, and RT PCR using these two primers on the adipose tissue of control and AdipoQ-LPL mice revealed that two isoforms exist (Fig. 7A). Sequence analysis of the PCR products revealed the presence of two distinct isoforms: full length Ccl19 and the short isoform, which is composed of exons 1, 3, and 4 (we note an anomaly in the way the gel ran affecting the migration of the isoforms in lane 5). We quantified the gel and found that *Ccl19* (exons 1,3,4) was more abundant in the adipose tissue of AdipoQ-LPL mice, and the full length form was more abundant in control mice, consistent with the microarray (Fig. 7B; P < 0.01). The exon structure and alternate splicing of *Ccl19* is shown in Fig. 7C. The *Ccl19* (1,3,4) splice isoform shifts the reading frame and causes a premature stop codon in the signal sequence such that no part of the secreted protein is made (Fig. 7D). Thus, the alternative isoform is completely inactive, and the regulation of *Ccl19* splicing is an effective mechanism for controlling *Ccl19* protein expression.

To determine whether SND1 regulates *Ccl19* splicing, we evaluated control and *Snd1* siRNA treated adipocytes that were induced to express *Ccl19* by TNFα treatment in Fig. 6. PCR analysis indicated that TNFα-treatment induced two Ccl19 isoforms (Fig. 7E inset). We then determined the expression level of each exon with exon specific primers, using *Ccl19* plasmid DNA to generate the standard curve, and determined the ratio of each exon to exon 1. As shown in Fig. 7E, the ratio of exon 2 to exon 1 was significantly decreased by *Snd1* siRNA (P < 0.05); *Snd1* siRNA treatment did not significantly reduce the exon 3 to exon 1 or the exon 4 to exon 1 ratios. Thus, SND1 promotes the inclusion of exon 2 into *Ccl19*, suggesting that reduced SND1 in vivo is responsible for the differential splicing of *Ccl19* observed in vivo (Fig. 7A,B). An analogous function has recently been shown for SND1 in cancer since it promotes the generation of splice isoforms of *CD44* that are oncogenic^29^.

### Metabolic phenotyping and insulin secretion

AdipoQ-LPL mice have improved glucose tolerance; however, the mechanism(s) are not completely identified^30^. An unexpected finding of the GSEA analysis of the microarray was the enrichment of the pancreatic β-cell hallmark gene set (shown in Table 3) in adipose tissue of AdipoQ-LPL mice (Table 2). Hypergycemia has been demonstrated to cause low levels of insulin expression in extra pancreatic tissues including adipose and liver tissues^31,32^. We therefore performed immunohistochemistry on the adipose tissue from the male mice on 10 days HFD that were characterized by micro array with insulin and pdx-1 antibodies, but were unable to identify insulin positive cells (not shown) and did not further characterize adipose tissue for the presence of β-cells by measuring gene expression of genes in the GSEA gene set (Table 3). Adipose tissue contains mesenchymal stem cells that can be induced to differentiate into pancreatic β-cells in vitro^31^, and it is possible that increased LPL expression can stimulate a pancreatic β-cell gene signature in these cells in adipose tissue. We hypothesized that if increased LPL in adipocytes of adipose tissue causes the secretion of factors that directs adipose mesenchymal stem cells towards a β-cell phenotype, then these factors could be released into the circulation to influence β-cell function in the pancreas. We therefore characterized glucose stimulated insulin secretion in AdipoQ-LPL mice to determine whether adipose tissue—β-cell crosstalk is a mechanism for the improved glucose tolerance of this mouse model.

**Table 3:** Genes in the pancreatic β-cell gene set.

Gene symbol^a^	Gene title
SYT13	Synaptotagmin XIII
MAFB	v-maf musculoaponeurotic fibrosarcoma oncogene homolog B (avian)
INSM1	Insulinoma-associated 1
INS	Insulin
PCSK1	Proprotein convertase subtilisin/kexin type 1
NKX6-1	NK6 transcription factor related, locus 1 (Drosophila)
DCX	Doublecortex; lissencephaly, X-linked (doublecortin)
CHGA	Chromogranin A (parathyroid secretory protein 1)
NEUROD1	Neurogenic differentiation 1
SLC2A2	Solute carrier family 2 (facilitated glucose transporter), member 2
SCGN	Secretagogin, EF-hand calcium binding protein
SST	Somatostatin
G6PC2	Glucose-6-phosphatase, catalytic, 2
FOXA2	Forkhead box A2
AKT3	v-akt murine thymoma viral oncogene homolog 3 (protein kinase B, gamma)
null	Null
GCK	Glucokinase (hexokinase 4, maturity onset diabetes of the young 2)
DPP4	Dipeptidyl-peptidase 4 (CD26, adenosine deaminase complexing protein 2)
PCSK2	Proprotein convertase subtilisin/kexin type 2
PAX4	Paired box gene 4
ISL1	ISL1 transcription factor, LIM/homeodomain, (islet-1)
GCG	Glucagon
ABCC8	ATP-binding cassette, sub-family C (CFTR/MRP), member 8
null	Null
PAX6	Paired box gene 6 (aniridia, keratitis)
null	Null
NKX2-2	NK2 transcription factor related, locus 2 (Drosophila)
IAPP	Islet amyloid polypeptide
PAK3	p21 (CDKN1A)-activated kinase 3
VDR	Vitamin D (1,25-dihydroxyvitamin D3) receptor
NEUROG3	Neurogenin 3
SPCS1	Signal peptidase complex subunit 1 homolog (S. cerevisiae)
LMO2	LIM domain only 2 (rhombotin-like 1)
STXBP1	syntaxin binding protein 1
PKLR	Pyruvate kinase, liver and RBC
SRP9	Signal recognition particle 9 kDa
SRPRB	Signal recognition particle receptor, B subunit

^a^The gene symbols and gene titles of the GSEA pancreatic β-cell gene set are indicated.

**Figure 8:**
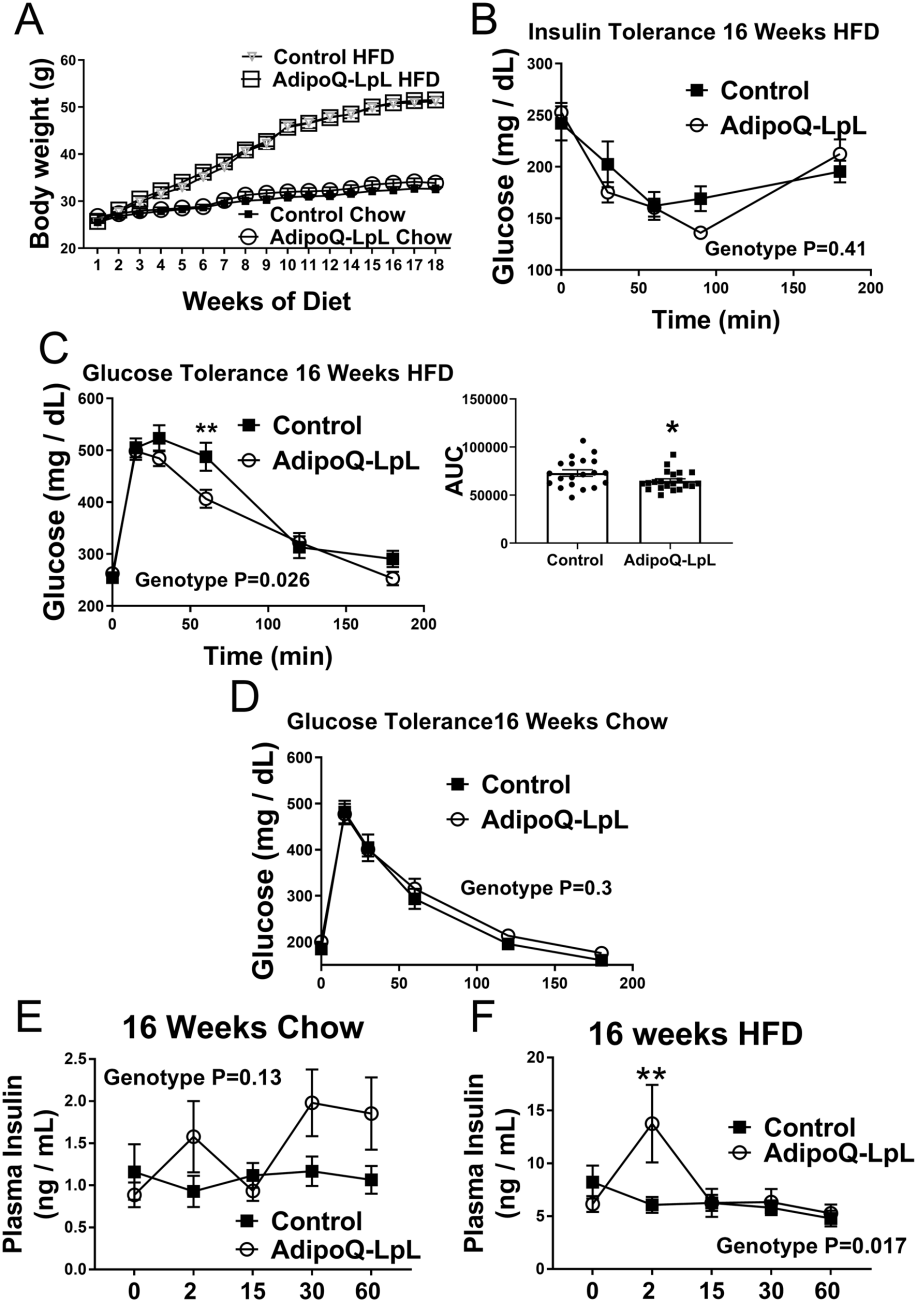
Glucose homeostasis and insulin secretion of control and AdipoQ-LpL mice. (**A**) Weight of the mice after switching to HFD (control n = 20; AdipoQ-LpL n = 21) or maintaining on chow (n = 12) starting at 8-weeks. (**B**) Glucose tolerance of control and AdipoQ-LPL mice after 16 weeks of HFD (control n = 20; AdipoQ-LpL n = 21). (**C**) Insulin tolerance of control and AdipoQ-LPL mice after 16 weeks of HFD (control n = 9; AdipoQ-LpL n = 9). Inset: analysis of area under the curve. (**E**,**F**) Plasma insulin levels after glucose injection of control and AdipoQ-LPL mice after chow or HFD for 16 weeks (control n = 11; AdipoQ-LPL n = 12; both cohorts 2 min glucose n = 6). Data were analyzed by RM 2 way ANOVA **P < 0.01; interaction P values for genotype are indicated in the figure.

We previously observed that if we place 5 week old AdipoQ-LPL mice on a high fat diet for 16 weeks, they have improved glucose and insulin tolerance^2^. However, in that model, the improved insulin sensitivity of the mice confounds studying insulin secretion. In a slightly different model, we have observed that if we place 8 week old AdipoQ-LPL mice on high fat diet for 18 weeks, they gain about 15 g more weight than 5 week old mice placed on HFD for 16 weeks (the original model^2^). The weights of mice maintained on chow or placed on HFD at 8 weeks are shown in Fig. 8A (we note that two cohorts of mice on HFD were studied; one cohort was characterized for glucose and insulin tolerance, and the second cohort was characterized for insulin secretion; the data shown is for both cohorts combined). AdipoQ-LPL mice that were placed on HFD for 16 weeks starting at 8 weeks of age do not have improved insulin sensitivity (Fig. 8B; P = 0.41), but still have improved glucose tolerance (Fig. 8C; P = 0.026). The results in Fig. 8C were also significant when area under the curve was analyzed (Fig. 8C inset). There was no improvement of glucose tolerance in AdipoQ-LPL mice on chow diet (Fig. 8D). Therefore, we studied insulin secretion during a glucose challenge in this mouse model since increased insulin secretion would explain the improvement in glucose tolerance. After 16 weeks on chow, there was no significant difference in insulin secretion (P = 0.13) between genotypes (Fig. 8E). At 16 weeks on HFD, the AdipoQ-LPL mice demonstrated increased insulin secretion (interaction P = 0.017), and this was due to an increase first phase insulin secretion (P < 0.01), which occurred 2 min after glucose injection (Fig. 8F).

### Summary and conclusion

Analyzing the adipose tissue transcriptional response to acute HFD challenge revealed two interesting mechanisms for the improved glucose homeostasis of LPL mice. First, SREBP2 is repressed in adipose tissue, and we found that SND1 is regulated by SREBP2. Thus SND1 is an important link between SREB2 and inflammatory responses. Second, pancreatic β-cell function is improved in AdipoQ-LPL mice since first phase insulin secretion is improved when diet induced obese mice are challenged with glucose. This is represents an important example of adipose tissue—pancreatic β-cell cross talk and provides a direct mechanism for the improved glucose tolerance of the AdipoQ-LPL mice.

## Discussion

We have characterized physiological responses and adipose tissue transcriptional responses of AdipoQ-LPL mice to a 10-day high fat diet challenge. Although, the AdipoQ-LPL mouse has increased food intake and increased resting energy expenditure after long term HFD^2^, no phenotype was apparent during the switch from chow to HFD in our calorimetry experiments. We characterized the transcriptional response to HFD in perigonadal adipose tissue since we observed significant changes in gene expression in that depot after long term HFD^2^. This analysis identified two interesting mechanisms by which increasing adipose tissue LPL reduces adipose dysfunction and improves glucose homeostasis. One mechanism involves the repression of the SREBP2 pathway, and further molecular analysis revealed that SND1 is induced by this pathway in gonadal adipose tissue, and that SND1 regulates CCL19 and promotes inflammatory cytokine production in vitro. The second mechanism involves improved pancreatic β-cell function. AdipoQ-LPL challenged with a high fat diet have increased first phase insulin secretion, which improves glucose tolerance.

A prominent aspect of the transcriptional response of AdipoQ-LPL mice is to repress enzymes involved in lipogenesis and cholesterol biosynthesis, which is a predicted response to increasing lipoprotein triglyceride hydrolysis and core uptake. We also found that genes regulated by PPARγ are upregulated in AdipoQ-LPL mice, which is also expected. In addition to this, GSEA revealed that several pathways thought to contribute to adipose dysfunction are repressed in AdipoQ-LPL mice. The primary mechanism likely involves increased lipoprotein core uptake into adipose and suppression of SREBP2 activity. SREBP2 is activated in adipocytes by high fat feeding to supply cholesterol for the growing plasma membrane and lipid droplet^6,33,34^. SREBP2 activation induces inflammatory cytokine expression during this process and others^6,35^, but the mechanisms are not completely understood. In this study, we find that the RNA binding protein SND1 is regulated by SREBP2 and contributes to inflammatory cytokine induction. It is likely that the down-regulation of SREBP2 in the adipose tissue of AdipoQ-LPL mice is a major contributor to the reduction in *Snd1* expression since our in vitro studies in 3T3L1 cells demonstrated that Snd1 is regulated by SREBP2. Indeed, a recent study showed that human SND1 is regulated by SREBP2^36^. Other pathways may also repress *Snd1* in AdipoQ-LPL mice since *Snd1* is also regulated by ER stress^37^, which GSEA indicated was down regulated in AdipoQ-LPL mice. *Snd1* is regulated by NFκB^38,39^, but we found that treatment of differentiated 3T3-L1 adipocytes with TNFa did not increase *Snd1* expression; the mechanism for this is unclear, but should be investigated in future studies.

SND1 is highly conserved and protects organisms from plants to eukaryotic cells from stress by its ability to interact with RNA and proteins^40^. By binding RNA, SND1 regulates mRNA expression at many levels including transcriptional coactivation, gene splicing, mRNA stability, and miRNA processing. SND1 is thus an interesting target of SREBP2 for a number of reasons. Studies in which SND1 is manipulated indicate that SND1 regulates the expression of genes involved in lipid biogenesis^41–44^ and very low density lipoprotein secretion in the liver^45^. Thus, SND1 may provide additional transcriptional regulation of genes involved in cholesterol biogenesis in response to SREBP2 activation, for example by regulating transcriptional activation and splicing. We found that SND1 regulates the splicing of *Ccl19*; however, a number of genes are alternatively spliced in AdipoQ-LPL mice (Table S2), and it will be interesting to examine the role of SND1 in this process. In addition, SND1 is a transcriptional cofactor for PPARγ and other transcription factors that are important in adipocytes. We did not observe repression of PPARγ regulated genes in response to reduced SND1. This could be due to stimulation of PPARγ by fatty acids generated by LPL; indeed, *Pck1* is induced in AdipoQ-LPL mice (Table S2).

In addition to regulating lipid biosynthesis SREBPs regulate immune responses^46,47^, and an example of this is the induction of TNFα and IL6 in adipocytes^6^, which is thought to be mediated by NFκB activation since these genes do not contain sterol response elements. Thus, the mechanisms by which SREBPs promote NFκB activation are unclear. We find that SND1 is induced by SREBP2 activation, and SND1 increases MCP1 and IL6 in TNFα-stimulated adipocytes, consistent with its ability to promote NFκB activity in hepatocytes^22^.

Our analysis of the microarray data uncovered an additional mechanism by which SND1 promotes inflammation: regulation of *Ccl19* alternative splicing. CCL19 is induced early in response to HFD challenge in adipose tissue^24^. Two studies suggest that mice deficient in the CCL19 receptor C–C motif chemokine receptor 7 (CCR7) are protected against diet induced obesity, adipose inflammation, and insulin resistance^24,48^; however, another study found no difference of CCR7 deficiency on metabolic homeostasis^49^. Here, we find that *Ccl19* is regulated by alternative splicing in vivo in AdipoQ-LPL mice, and our investigation of the mechanism in vitro revealed that SND1 promotes the inclusion of exon 2 into *Ccl19* mRNA. Similar to this, SND1 has recently been shown to promote the inclusion of an exon with a weak splice site into *Cd44* to promote tumorigenesis^29^. Thus, regulation of alternative splicing is an important function of SND1, and it will be interesting to investigate the alternative splicing of other genes that we have identified in this study including metabolic genes and genes involved in adipose dysfunction.

An unexpected finding from the GSEA analysis was the identification of the pancreatic β-cell hallmark in gonadal adipose tissue. The mRNA level of insulin was very low in adipose tissue, and it was thus unlikely that insulin secretion from adipose tissue, if any, was having an impact on glucose homeostasis in the AdipoQ-LpL mouse. We did not pursue the mechanism by which increasing LPL in adipocytes results in the pancreatic β-cell hallmark in gonadal adipose tissue. We propose that increased adipocyte LPL was increasing the expression level of protein factors or affecting lipid or metabolite levels that affect mesenchymal stem cell differentiation pathways. For instance, betacellulin was identified in Table S1, and it is known that betacellulin stimulates the formation of insulin secreting cells^50^. We propose a similar model for adipose tissue—pancreatic beta cell crosstalk where increased LPL expression in adipocytes causes the release of proteins, bioactive lipids, or other factor that affect β-cell function in the pancreas. Thus an important goal is to identify these factors and determine their effects in vivo and on islets ex vivo. Our studies on insulin secretion revealed that AdipoQ-LPL mice have increased first phase insulin secretion. It is interesting that only first phase insulin secretion was affected. There are thought to be two pools of insulin granules, a reserve pool and a readily releasable pool^51^, and it will be important to dissect the mechanism by which first phase insulin secretion is increased in the AdipoQ-LPL mouse model.

We initially hypothesized that increasing expression of LPL in adipocytes would reduce lipotoxicity and improve insulin sensitivity and glucose homeostasis. However skeletal muscle and liver did not have reduced diacylglycerol or ceramide when the AdipoQ-LPL mice were placed on HFD, and this is likely due to the fact that the adiponectin promoter only modestly increases adipose tissue LPL mRNA levels^2^. We did not isolate pancreatic islets in this study, and it is possible that there is reduced pancreatic β-cell lipotoxicity, which would improve β-cell function^52–54^. Other mechanisms possible for enhanced β-cell function would be that proteins are secreted from adipose tissue that affect β-cell function. Cross talk between adipose tissue and the pancreas has recently begun to be explored. Adiponectin and leptin both have positive effects on pancreatic β-cells by reducing lipotoxicity, and adipsin has been recently demonstrated to have positive effects on β-cells and insulin secretion^55,56^. It is thus possible that AdipoQ-LPL mice increase these or other factors such as lipids or metabolites that, in turn, affect β-cell function in the setting of HFD-induced obesity. Indeed, we previously observed increased adiponectin in the serum of AdipoQ-LPL mice^30^. Identification of the molecules mediating adipose tissue- β-cell crosstalk in this mouse model is an important future goal.

There are important limitations to this study. First, the microarray used was not as comprehensive as RNA seq, and thus certain genes and pathways may be over or under represented. Second, analysis of the microarray data by GSEA indicated enrichment of a β-cell hallmark in the adipose tissue of the AdipoQ-LPL mice. However, we were not able to confirm the presence of beta cells in adipose tissue, and the source of this signal is unknown. We utilized the GSEA result only to hypothesize adipose tissue—beta cell cross talk. This hypothesis relies on the tissue specific expression of LPL by the adiponectin promoter. We have not observed transgene expression in tissues besides adipose^2^ as expected, but have not tested all tissues and cell types.

In conclusion, analyzing the transcriptional response of AdipoQ-LPL mice has revealed that several pathways that contribute to adipose tissue dysfunction are repressed including the SREBP2 pathway. *Snd1* gene and protein expression are increased by SREBP2 activation, and SND1 promotes inflammatory cytokine and chemokine expression, including regulating the expression of the active *Ccl19* splice isoform. SND1 may be an important SREBP2 target due to SND1’s ability to control transcriptional responses at multiple levels. Finally, this study suggests that increasing LPL in adipose tissue may stimulate signaling pathways between adipose tissue and pancreatic β-cells, which could have a significant impact on glucose homeostasis.

## Materials and methods

### Animal studies

All of the studies involving mice were approved by the University of Kentucky Institutional Animal Care and Use Committee. The mice were housed and given high fat diet (HFD) (60% kcal from fat; D12492; Research Diets, New Brunswick, NJ, USA) at the indicated age as previously described^2^. Body composition was determined before and after the indirect calorimetry study with an Echo MRI system (Echo Medical Systems, Houston, TX, USA). Mice were evaluated by indirect calorimetry (TSE systems, Chesterfield, MO, USA). Wild-type female C57B6/J mice (The Jackson Laboratory, Bar Harbor, ME, USA) were used for backcrossing. Euthanasia was performed on conscious mice using carbon dioxide. All methods were performed in accordance with relevant guidelines and regulations. The study is reported in accordance with ARRIVE guidelines.

### Cell culture and treatments

3T3-L1 cells (ATCC) were cultured in DMEM (Life technologies, Grandland, NY, USA) with 10% new born calf serum (TCB, Tulare, CA, USA) and differentiated into adipocytes as follows. Cells were seeded at density of 8 × 10^4^/well in 6-well plates, and the medium was changed every day until they became confluent. 48-h after becoming confluent, the medium was replaced with differentiation medium (DMEM with 10% FBS (TCB, Tulare, CA, USA), 1 μM dexamethasone (Sigma, St. Louis, MO, USA), 0.5 mM methylisobutylxanthine (Sigma, St. Louis, MO, USA), 1 μg/mL Insulin (Novo Nordisk, Bagsvaerd, Denmark). After 4-days, differentiation medium was replaced with adipocyte maintenance medium (DMEM with 10% FBS and 1 μg/mL Insulin), which was replaced every 2-days. For the cholesterol depletion experiments, after 10-days of differentiation, the adipocytes were washed with PBS and then incubated in medium supplemented with 10% delipidated FCS (J65182; Alfa Aesar, Tewksbury, MA, USA), 50 μM sodium mevalonate (Sigma, St. Louis, MO), and 50 μM sodium compactin (Sigma, St. Louis, MO) for 16-h. After this, the adipocytes were incubated in the same medium supplemented with either ethanol (vehicle control) or 1 μg/ml cholesterol (Sigma) for 4-h. SREBP2 was detected with rabbit anti-SREBP2 (10007663; Cayman Chemical Company, Ann Arbor, MI, USA); SND1 was detected with rabbit anti-SND1 (4200470; Sigma); and actin was detected with mouse anti-actin (A1978; Sigma). The immunoblots were imaged and quantified with secondary antibodies and an Odyssey imaging system (LI-Biosciences, Lincoln, NE) as described^57^ or a ChemiDocTM MP imaging system Bio-RAD (Hercules, CA, USA) using Clarity Western ECL Substrate ECL (Cat # 170-5060; Bio-RAD). For the siRNA experiments, after 8-days of differentiation, the adipocytes were transfected with equivalent amounts of control (scrambled), mouse *Snd1* SMARTpool siRNA (56463; Dharmacon, Lafayette, CO), or mouse Srebp2 SMARTpool siRNA (L-050073-01;Dharmacon) using Lipofectamine RNAiMAX (Thermo Fisher Scientific) according to the manufacturer’s instructions. For the SND1 TNFα experiment, after 48-h, the adipocytes were treated with 10 ng/mL tumor necrosis factor-α (TNFα) (R&D systems, Minneapolis, MN) for 4-h as indicated, and the adipocytes were harvested for mRNA analysis.

### Collagenase digestion of adipose tissue

Perigonadal adipose tissue was immediately minced and incubated in 1 mg/mL type I collagenase in DMEM for 30-min at 37 °C with agitation. The adipose was centrifuged at 600*g* for 10-min and the floating adipocytes and stromal vascular fraction isolated. Actin was detected with mouse anti actin, and SND1 was detected with mouse anti-SND1 (SAB4200504 (Sigma).

### Microarray

Total RNA from perigonadal fat was isolated with an RNeasy lipid tissue mini kit (74804, Qiagen, Hilden, Germany) using Qiazol™ (Qiagen) as the adipose tissue lysis reagent. The RNA concentration and integrity was then characterized with an Agilent Bioanalyzer and analyzed by affymetrix mouse exon 2.0 arrays, which measured exon expression of 41,346 transcripts, at the University of Kentucky microarray facility. Data analysis was performed with Partek software on RMA-normalized data that was log2-transformed. Additional analysis was performed with NIH David and gene set enrichment analysis. The data discussed in this publication have been deposited in NCBI's Gene Expression Omnibus and are accessible through GEO Series accession number GSE87661 (https://www.ncbi.nlm.nih.gov/geo/query/acc.cgi?acc=GSE87661).

### Gene expression

Gene expression was determined by real time reverse transcriptase polymerase chain reaction as previously described^58^. Standard curves were made from a pool of the cDNA, and gene expression was normalized to 18S. This allows for the measurement of relative changes in gene expression within the indicated experiment. The primer sequences are in Table 4.

**Table 4:** Primers.

Gene^a^	Forward primer	Reverse primer
*Snd1*	GGG ACG AGA GTA TGG GAT GAT	GCC TTC TGC AAC TAG CGA CT
*Ldlr*	AGT GGC CCC GAA TCA TTG AC	CTA ACT AAA CAC CAG ACA GAG GC
*Lss*	TCG TGG GGG ACC CTA TAA AAC	CGT CCT CCG CTT GAT AAT AAG TC
*Insig1*	TGT CGG TTT ACT GTA TCC CTG T	GTT GAT GCC AAC GAA CAC GG
*Lrrc4*	GAG GAG CTT GAG ATG TCA GGG	CCA GTC CGT CAA AAG CAT TCC
*Ccl19*	GGG GTG CTA ATG ATG CGG AA	CCT TAG TGT GGT GAA CAC AAC A
*Mcp1*	ACA CTG GTT CCT GAC TCC TCT	ATT AGA TTC GGT TTA ATT GGC CC
*Il6*	CCA AGA GGT GAG TGC TTC CC	CTG TTG TTC AGA CTC TCT CCC T
*Ccl19 e1*	CAC TCA CTC TCT GTG GCC TG	CAG AGA ACC AGC AGG CTG AA
*Ccl19 e2*	GAA GAC TGC TGC CTG TCT GT	ACC CTG CAG CCA TCT TCA TT
*Ccl19 e3*	GTT CAC CAC ACT AAG GGG CT	CTT CGG ATG ATG CGA TCC AC
*Ccl19 e4*	ATC ACT CTG GCC CAG GAA AC	CTT GGC TGG GTT AGG TCT GG
*Ccl19 e1-e4*	CAG TCA CTC CCC TGT GAA CC	CTT GGC TGG GTT AGG TCT GG
*18S*	GTAACCCGTTGAACCCCATT	CCATCCAATCGGTAGTAGCG

^a^The sequences of primers used for realtime PCR are indicated. In addition, we designed primers to amplify the four exons of *Ccl19* (e1, e2, e3, and e4). The primers used to identify *Ccl19* isoforms (*CCl19* e1-e4) by PCR in adipose tissue are also indicated.

### Analysis of Ccl19 splicing

For the analysis of *Ccl19* splicing in adipose tissue, cDNA was synthesized with QuantiTect Reverse Transcription Kit (Qiagen) from total RNA isolated from control and AdipoQ-LPL mice perigonaldal adipose tissue. PCR was performed with above cDNA as template and primers located in exon 1 and exon 4 of *Ccl19* (Table 1). The PCR products were resolved in agarose gels, imaged and quantified with an Alphaimager HP system (proteinsimple, San Jose, CA, USA), isolated, and sequenced. For the analysis of *Ccl19* splicing in vitro, exon specific primers (Table 4) were used for realtime PCR on cDNA from control and SND1 siRNA transfected 3T3-L1 adipocytes with TNFα 4-h; mouse *Ccl19* plasmid DNA was used for the standard curve.

### Energy metabolism studies

The study of mice in TSE calorimetry chambers was performed as described^2^ except that the mice were studied on chow and then switched to a 60% high fat diet during the study. The indirect calorimetry data were analyzed by ANCOVA as previously described^2^.

### Mouse metabolic phenotyping

Glucose and insulin tolerance was performed as described^2^. Insulin secretion was evaluated as follows. Blood (10 μL) was isolated from the mouse tail vein at the indicated time after intraperitoneal glucose injection (10 days: 1 g/kg; 16 weeks: 2 g/kg) in a tube containing 1 μL EDTA. Plasma insulin was measured using an ultrasensitive mouse insulin ELISA kit (#90080; Crystal Chem, IL) according to the manufacturer’s instructions.

### Statistical analysis

Data for the two groups of mice were analyzed by an unpaired, two-tailed student T-test. Multiple comparisons were analyzed by ANOVA and Tukey’s post hoc analysis using Graph Pad Prism 5 and 7. Statistical significance was set at a P value less than 0.05.

## Supplementary Information

Supplementary Figures.

Supplementary Tables.

## Data Availability

The datasets generated during and/or analyzed during the current study are available from the corresponding author on reasonable request.
