# Supplementary material for: Improved β-cell function leads to improved glucose tolerance in a transgenic mouse expressing lipoprotein lipase in adipocytes
Source: Sci Rep. 2022 Dec 24;12:22291. doi: 10.1038/s41598-022-26995-1 (PMC9789969; doi:10.1038/s41598-022-26995-1)
Supplement: Supplementary file 1 — Supplementary Figures. [file 41598_2022_26995_MOESM1_ESM.pdf]

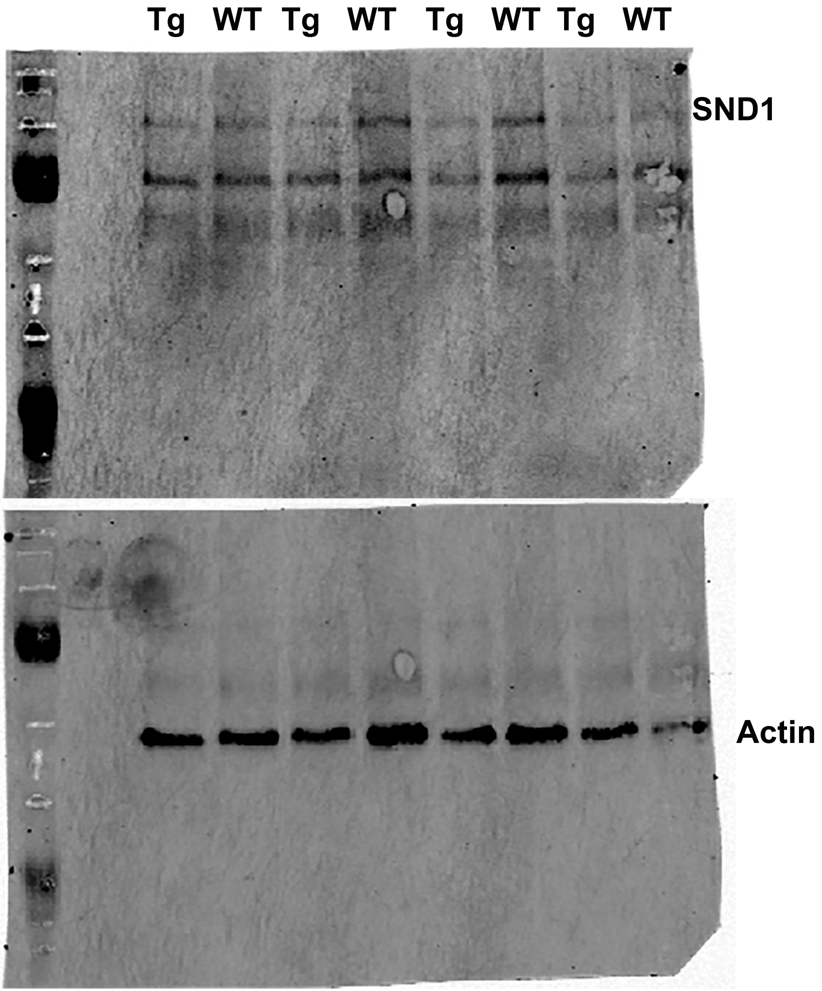

Figure S1. Full uncropped gels for Figure 4. Top) Immunoblot of adipose tissue for SND1. Bottom) The blot was reprobed for actin.

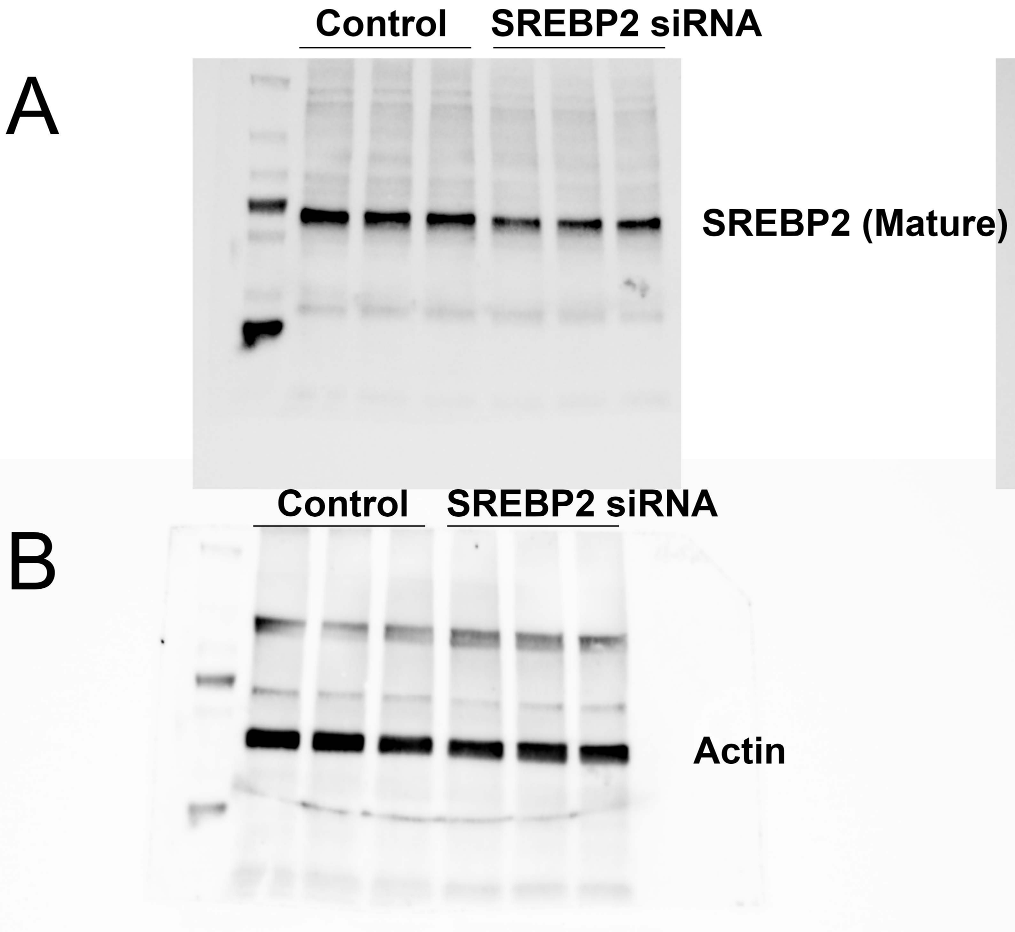

Supplemental Figure 2. Full immunoblots of siRNA mediated SREBP2 knockdown in 3T3L1 cells. Differentiated 3T3L1 adipocytes were treated with control or SREBP2 siRNA. A) immunoblot of SREBP2. B) Immunoblot of Actin.

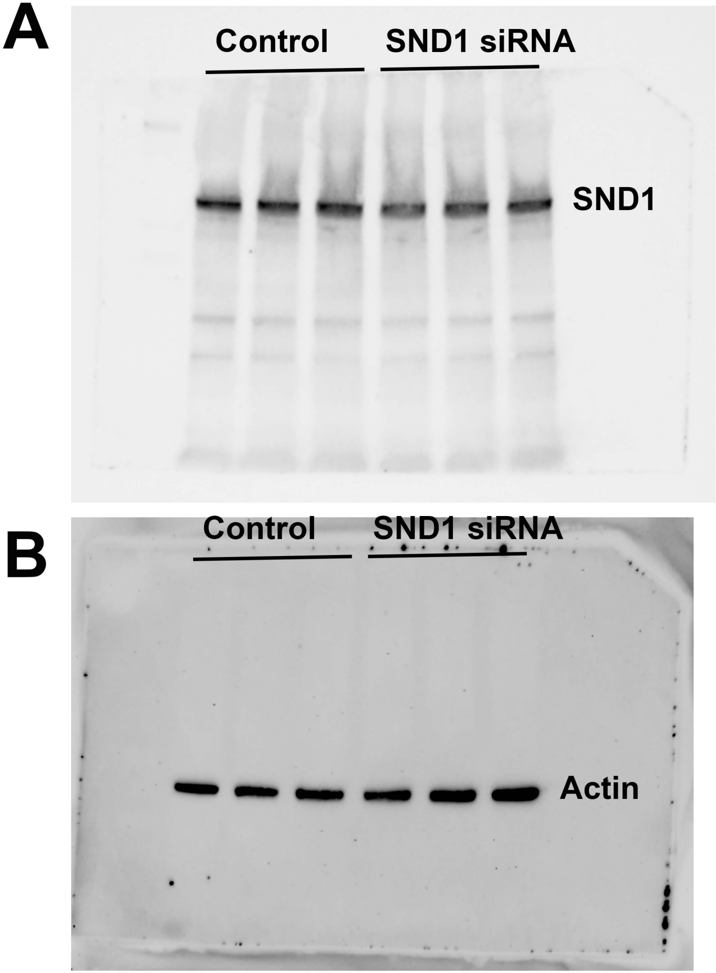

Supplemental Figure. Full immunoblots of siRNA mediated SND1 knockdown in 3T3L1 cells. Differentiated 3T3L1 adipocytes were treated with control or SND1 siRNA. A) immunoblot of SND1. B) Immunoblot of Actin.

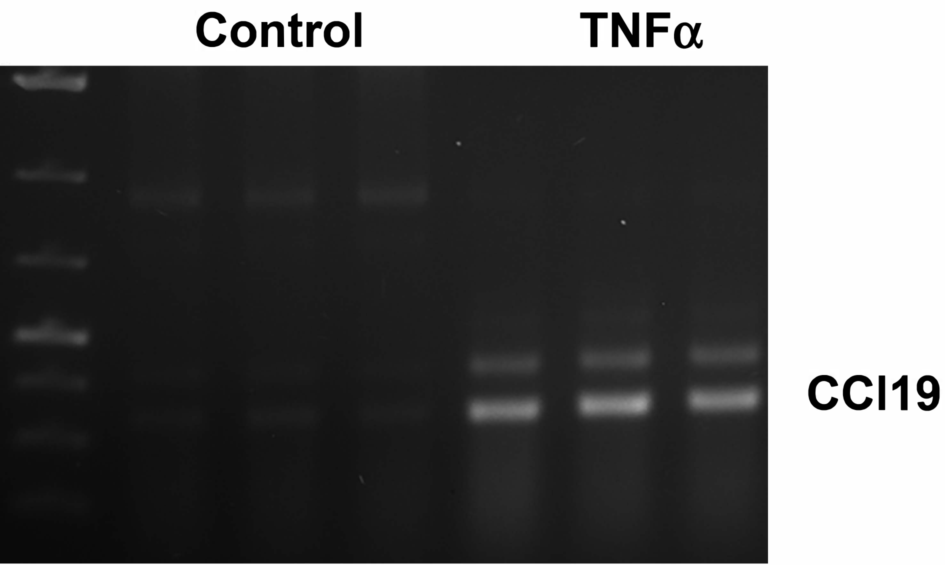

Figure S4. Uncropped gel for Figure 7E inset.
